# European Union implementation of Article 5.3 of the Framework Convention on Tobacco Control

**DOI:** 10.1186/s12992-018-0386-1

**Published:** 2018-08-02

**Authors:** Benjamin Hawkins, Chris Holden

**Affiliations:** 1 Faculty of Public Health and Policy, London School of Hygiene and Tropical Medicine, 15-17 Tavistock Place, London, WC1H 9SH UK; 2 Department of Social Policy and Social Work, University of York, York, UK

**Keywords:** Tobacco control, Tobacco industry, Framework Convention on Tobacco Control (FCTC), Article 5.3, European Union (EU), European Commission, European Parliament

## Abstract

**Background:**

Article 5.3 of the World Health Organization’s Framework Convention on Tobacco Control (FCTC) requires Parties to the agreement to take proactive measures to protect health policy from the vested interests of the tobacco industry. Parties to the FCTC are required to submit periodic reports to the Convention Secretariat on the efforts undertaken to implement it. Previous analyses of national compliance with the FCTC suggest that Article 5.3 implementation is piecemeal and insufficient in many contexts, with governments relying on general transparency and other existing policies for the purpose of Article 5.3 implementation. No in-depth study of Article 5.3 compliance within the European Union (EU) – a signatory to the Convention – has been undertaken. This study seeks to assess the extent of Article 5.3 compliance in European Union institutions, through an analysis of the mechanisms in place in the European Commission and European Parliament. It analyses EU documents relevant to Article 5.3 compliance, as well as semi-structured interviews with policy actors in the EU institutions and the field of tobacco control.

**Results:**

As with many national governments, Article 5.3 compliance within EU institutions is partial and incomplete. Much of the compliance activity cited in EU reports is derived from general codes of conduct for EU staff and the Juncker Commission’s transparency agenda. Interview respondents reveal widespread lack of knowledge about the existence of the FCTC and Article 5.3 amongst key policy actors across the institutions. Within the Commission policies vary greatly between Directorates General, and issues surrounding the conceptualisation of the role of Members of the European Parliament affect implementation in that context. While there is growing awareness of the issue in both the Commission and the Parliament, in large part as a result of the experience of lobbying over the Tobacco Products Directive, there remains considerable resistance in both institutions to further substantive action to implement Article 5.3.

**Conclusions:**

We recommend that a binding and comprehensive policy and code of conduct, specifically designed for the implementation of Article 5.3 and based on the World Health Organization’s guidelines, be created to cover the activities of all employees of all EU institutions. Crucially, such guidelines would need to deal explicitly with third parties acting for the tobacco industry.

## Background

Article 5.3 of the World Health Organization’s (WHO) Framework Convention on Tobacco Control (FCTC) requires governments to take measures to protect health policy “from commercial and other vested interests of the tobacco industry” [1]. Moreover, Parties to the FCTC are required to submit periodic reports to the Convention Secretariat on the efforts undertaken to implement its provisions, including Article 5.3, via responses to a standardised questionnaire, which are publicly available online. In 2008, the Conference of the Parties (COP) to the FCTC adopted a series of guidelines and recommendations for the implementation of Article 5.3 [2]. Consequently, scholars seeking to assess compliance with Article 5.3 have both a data source and a set of criteria against which to assess governments’ activities in this area. In the first large-scale assessment of Article 5.3 compliance, Fooks et al. [3] used these data to undertake a comparative, quantitative analysis of compliance across Parties to the agreement. That study found that Article 5.3 compliance was highly selective, with only 16% of guideline recommendations reviewed being implemented across all Parties. Moreover, 83% of Parties that had taken some action under Article 5.3 had implemented less than a third of the guidelines. There are few other systematic evaluations of Article 5.3 implementation, although what little work there has been confirms Fooks et al.’s analysis that implementation has been only partial [4–7].

The present article seeks to build on the previous, macro-level, analysis of Article 5.3 compliance conducted by Fooks et al. [3], through an in-depth, qualitative case study of the European Union (EU). Fooks et al.’s analysis did not include the EU since, as a supranational organisation, it is not strictly comparable with state Parties to the FCTC. Nevertheless, as a Party to the FCTC, independent from its member states, the EU is bound by its provisions and must undertake separate compliance activities. Fooks et al. [3] also concluded that in-depth qualitative case studies, drawing on key-informant interviews, were necessary to better understand Article 5.3 implementation, in part since widespread misreporting in FCTC Party reports suggests that many public officials have a weak understanding of Article 5.3. Additionally, many Parties were found to implement Article 5.3 through existing generic guidelines on corporate lobbying and transparency, while others implemented Article 5.3 through working norms, rather than through explicitly codified guidelines, making it difficult to assess how effective compliance efforts are in practice.

The intensity with which transnational tobacco companies (TTCs) have lobbied EU institutions, most recently over the 2014 revision of the EU’s Tobacco Products Directive (TPD), has been well documented [8–10]. Such lobbying appears to have had significant success in influencing policy outcomes [11]. The EU represents the most ambitious and advanced form of institutionalised transnational governance in history, based on a series of (periodically revised) foundational treaties, secondary legislation and jurisprudence of the Court of Justice of the European Union (CJEU), collectively known as the *acquis communautaire*. Given this complexity, implementation of Article 5.3, and the evaluation of that process, are extremely challenging. Consequently, we focus here on the main supranational legislative and policy-making bodies – the European Commission and the European Parliament (EP) – and the Article 5.3 guideline recommendations that are most relevant to them, but take note of the implications of our analysis for other EU bodies where relevant. The Council of the European Union (‘the Council’) and the European Council (on the distinction see [12]), are more difficult to analyse in this context as, in representing the interests of member states, their activities involve national-level policy actors. Thus a full exposition of Article 5.3 compliance in the Council and European Council would require analysis of policies and mechanisms in place at the member state level, and is beyond the scope of the current article. These bodies do, however, have a joint permanent secretariat, employed directly by the EU and tasked with the organisation and administration of Council business, although these are dwarfed in size by the Commission. Functionaries employed in these institutions are in a position very similar to Commission officials in regard to Article 5.3 compliance. Thus, recommendations we put forward in relation to EU officials are applicable not just to the Commission, but to those in the Council secretariat and other EU agencies and institutions (e.g. the CJEU, the European Central Bank, the European Ombudsman, the Committee of the Regions and the Economic and Social Committee).

We focus on WHO Article 5.3 guideline recommendations 1.1–1.2 on awareness raising, 2.1–2.2 on limiting interactions with the tobacco industry, 4.1–4.11 on the avoidance of conflicts of interest, and 5.1–5.5 on transparency (see Table 2). We do not focus on recommendations 3.1–3.4 on the rejection of industry partnerships, or on recommendations 6.1–6.4 on the denormalisation of industry “corporate social responsibility” activities, although we note in this context that important questions have been raised about whether the EU’s partnership agreements with the largest TTC’s on illicit trade are compliant with Article 5.3 [13, 14]. We also do not focus on WHO recommendations 7.1–7.3 on preferential treatment of the tobacco industry, or on recommendations 8.1–8.3 on state-owned tobacco industries, since these are less relevant to the EU institutions.

The article proceeds as follows. First, we discuss our methods. Second, we briefly describe the principal functions and processes of the Commission and the EP for a non-specialist audience. We then discuss generic transparency processes in the EU, as a necessary context for the presentation of our results. Third, we present our results, beginning with an analysis of documents related to the EU institutions in general, before proceeding to analyse Article 5.3 implementation specifically in the Commission and the EP. Finally, we discuss the results and conclude.

## Methods

Our methods consisted of two steps: first an evaluation of documents and, second, a series of semi-structured interviews with key informants. We first reviewed a number of publicly available documentary sources relating to both generic practices on transparency and ethical behaviour within EU institutions, and to the specific implementation of Article 5.3. The latter included EU reports to the FCTC Secretariat and documents relating to the European Ombudsman’s inquiry into a complaint against the Commission that it had failed to properly implement Article 5.3. The former included documents setting out generic rules and guidelines on ethical behaviour for EU officials, including the *Staff Regulations* for EU officials, the *European Code of Good Administrative Behaviour*, the *Code of Conduct for Commissioners* and the Commission’s *Practical Guide to Staff Ethics and Conduct*, many of which were cited by the Commission in relation to Article 5.3 compliance in its submissions to the FCTC Secretariat and to the European Ombudsman. However, given the lack of governmental understanding of Article 5.3, and the requirements for its full implementation previously identified [3], it is conceivable that the Commission (like some national governments) perhaps underreported relevant measures in place. Therefore, we did not rely on the self-reporting of the Commission in the identification of relevant policies, and instead pro-actively searched for relevant documentation beyond those cited in reports to the FCTC Secretariat. This led, for example, to the identification of the *Code of Conduct for Members of the European Parliament with Respect to Financial Interests and Conflicts of Interest* [15], and the *Guide to the Obligations of Officials and Other Servants of the European Parliament - Code of Conduct* [16], which were not referenced in Commission reports to the FCTC Secretariat. Below, we analyse documents which refer to the EU overall, to the Commission and to the EP in the relevant sections. A larger volume of documents was identified pertaining to the Commission than to the EP. In part, this reflects the case brought to the European Ombudsman against the Commission in relation to Article 5.3 and the transparency agenda launched by the Commission in the wake of this. As will be discussed below, it suggests also a piecemeal and incomplete approach to Article 5.3 compliance, as well as to broader issues of conflict of interest, across the institutions.

Second, we conducted a series of qualitative, semi structured interviews (*n* = 32) between February 2015 and December 2016 with policy actors in Brussels and Dublin, engaged in, or with knowledge of, tobacco-control issues. Interviews were conducted to address the potential drawbacks, discussed above, of relying on self-reporting by actors who may have limited understanding of the requirements of Article 5.3 compliance. Moreover, they allowed us to examine and probe compliance measures and knowledge of Article 5.3 within the institutions in greater detail. This allowed us to develop a more nuanced understanding not just of activities and structures in place, but the underlying norms governing institutional approaches to the FCTC and Article 5.3, and to conflicts of interest more broadly. As well as specific questions about Article 5.3 and about generic policies and practices relevant to Article 5.3 implementation, interviews focused on two key policy areas relevant to tobacco control and tobacco company policy influence at the EU level that had been central to the work of the Commission and the EP in the period during and immediately prior to the interviews: the passage of the EU’s revised TPD, and trade negotiations relating to the Trans-Atlantic Trade and Investment Partnership (TTIP) with the US and the Comprehensive Economic and Trade Agreement (CETA) with Canada. Tobacco industry lobbying in EU institutions was extensive during the passage of the TPD [8, 9], while external trade and investment agreements are a key responsibility of the Commission and have been identified as offering tobacco companies wide-ranging opportunities for policy influence [3, 17, 18]. Identification and recruitment of interview respondents was via purposive (from documentary reviews) and snowball sampling (from interviewees) until we had reached saturation [19]. Access was sought via email and telephone contact and interviewees gave explicit informed consent for participation and interview recording, indicating the level of anonymity they requested for their responses. Respondents could indicate whether they could be identified by name, organisation or in more general ways, which would avoid them being identified personally, i.e. by organisation type or sector. Interviews were generally around an hour in length, although some lasted up to an hour and a half. A wide range of actors agreed to be interviewed, giving multiple perspectives on EU tobacco control, including representatives from NGOs and civil society as well as the relevant EU institutions. A breakdown of respondents by sector is provided in Table 1 below. Due to ethical issues specific to engaging with the tobacco industry, no interviews were conducted with industry actors [20]. Interviews in Dublin were included due to the vital role played by the Irish government in the passage of the TPD during its presidency of the Council of the European Union in the first semester of 2013, and the wider leading role played by Ireland in European tobacco control. Interview responses were triangulated with the documents discussed above. Interview recordings were transcribed, anonymised and stored on a secure drive.

**Table 1:** Interview Respondents by Sector

	Brussels	Dublin
Officials (EU/ national civil servants)	4	0
Parliamentarians		
(Members of the European Parliament/ Oireachtas, and their advisors)	4	4
Public health NGOs	10	6
Journalists	1	3
Total	19	13

The first author coded interviews using Nvivo data analysis software through an iterative, two-stage process of analysis. Initial codes were derived from the WHO’s Article 5.3 guidelines. Further codes were identified inductively through an initial reading of interview transcripts. These were added to, and used to refine, the initial list of codes in liaison with the second author. This code list was then used to code the transcripts systematically in the second reading via Nvivo. This allowed for the coding of topics and themes not directly related to, and not directly corresponding with, the implementation guidelines. Inductive themes identified related to issues such as awareness and understanding of Article 5.3, how policies and ethical guidelines are implemented in practice, and the norms and ‘culture’ of EU institutions that influence the latter. The presentation of data below is not primarily organised around method, although document analysis generally precedes the presentation of interview data. Rather our analysis is organised primarily around the WHO Article 5.3 implementation guidelines and the institutions examined. In each section we draw on both documentary and interview data to build a detailed picture of compliance activities, drawing on the principle of triangulation. Given the relative lack of documents relating to the EP, this section draws more substantially on interview data. To ensure maximum anonymity for respondents, our default approach is to attribute all quotes by sector only, except where additional details were essential to contextualise the statement or underline its reliability, and permission for more precise attribution was given by respondents. Ethical approval for this study was granted by the relevant ethics committees at the authors’ institutions. Below, we provide a brief overview of policy-making processes in the EU and set out the development of generic transparency rules and practices in the EU institutions. This provides a necessary context for the subsequent analysis of measures explicitly targeted at Article 5.3 compliance.

## Policy making in the European Union

Any evaluation of the implementation of Article 5.3 must take into account the specific political and institutional context under consideration. Here, we briefly set out the main aspects relevant to the Commission and the EP. The Commission is a unique political institution and a key body in the EU system, performing core policy, legislative, monitoring and enforcement functions under EU law (see [21]). At the apex of the Commission hierarchy is the College of 28 Commissioners, drawn from each of the member states and assigned an individual policy portfolio (e.g. Health, Single Market, etc.). Commissioners are supported by their personal staff known as their Cabinet, with the Heads of Cabinet playing highly influential roles in Commission business. Below this are the Directorates General (DGs), Directorates and Units into which the Commission bureaucracy is organised. DGs are organised in terms of policy portfolios which map broadly onto those of the College. The Commission is designated the “guardian of the treaties” and is tasked with ensuring EU law and policies are implemented. It administers and distributes the EU budget, and is responsible for undertaking negotiations with third parties, including international accords such as the FCTC. Whilst EU legislation is passed by the EP and the Council (on an equal footing under the "ordinary legislative procedure", which is the default legislative procedure in the EU; see [22]) they can do so only following a proposal from the Commission, which has the sole right of initiative. As such, the Commission plays a vital agenda-setting and policy-development role and is a key target for lobbyists and policy advocates.

The role of the EP is to represent the interests of EU citizens via directly elected Members of the European Parliament (MEPs). It plays a central role in the legislative and budgetary procedures and has the right of approval on the appointment of the Commission and in external, third-party agreements (see [23]). As with national parliaments, much of the work of the EP in scrutinising legislation is undertaken by specialist committees prior to votes in plenary. For example, the Health and Environment Committee (ENVI) was the lead committee for the TPD. The EP is structured around party groupings, with the largest two being the Centre-Right European People’s Party (EPP) and the Centre-Left Progressive Alliance of Socialists and Democrats (S&D). In committees, each piece of legislation is assigned a *rapporteur* with responsibility for the passage of the bill from one of the party groupings, with others assigning shadow *rapporteurs* to present their group’s interests. For the 2014 TPD, the rapporteur in the ENVI committee was UK MEP Linda McAvan (S&D), shadowed by Germany’s Karl-Heinz Florenz (EPP).

### Transparency in the EU institutions

Concerns over lobbying within the EU institutions have led to a series of measures being adopted, which aim at increasing the transparency of contacts with external actors. Initially this occurred on a voluntary basis, with the EP setting up a transparency register in 1995, followed by the Commission in 2008, before these were consolidated in a single register via an inter-institutional agreement in 2011, itself modified by a further such agreement in 2014 [24]. The inadequacies of the existing voluntary regime were never clearer than during the ‘Dalligate’ scandal which surrounded the Commission in 2012 and led to the resignation of the Maltese Health Commissioner, John Dalli, following allegations he had met tobacco industry actors [25].The Juncker Commission’s “transparency agenda”, under the direction of First Vice President Frans Timmermans, was a response to criticisms of the Commission’s openness to lobbyists in the aftermath of the Dalli affair. In 2014, the Juncker Commission’s Political Guidelines included a range of measures designed to increase transparency and oversight of the Commission’s activities and its engagement with external actors, including corporations [26]. More specifically, this has seen specific disclosure rules for documents relating to the TTIP negotiations in November 2014 [27] (discussed below), new rules adopted for the open appointment of external expert groups in May 2016 [28], and additional requirements for document releases as part of the Inter-Institutional Agreement on Better Law Making in September that year [29].

Of most relevance for the implementation of Article 5.3, however, are the measures in place to monitor policy influence by external actors through the registration of lobbyists. Criticism of the incomplete and porous nature of the current voluntary regime, and the experience of the TPD process, have led to proposals for a “mandatory” Transparency Register covering the EP, Council and Commission, put forward by the Commission in September 2016 [30]. This aimed to build on and extend the previous regime, covering just the Commission and the EP. Under the proposed Inter-Institutional Agreement, all external actors seeking to engage the institutions in an attempt to influence the content or implementation of policy would need to be included on a central register of lobbyists in order to be able to meet with the highest ranking officials. Within the Commission this would include Commissioners, Heads of Cabinet and Directors General. Within the EP, meetings with MEPs, the Secretary General, Directors General and Secretaries General of political groups as well as access to Parliamentary buildings would be limited to registered lobbyists. As regards the Council, the proposed agreement would cover meetings with Ambassadors and Deputy Ambassadors of the current or forthcoming Presidency, the Council’s Secretary General and Directors General. The proposed agreement grants leeway to individual institutions to extend the registration requirement to meetings with other officials.

At the same time, it sets out a range of organisations and types of interactions which are explicitly excluded from the registration requirements. These include social partners, religious bodies, representations made by citizens in an individual capacity, national and sub-national agencies of member states, third-party states and international organizations. As will be discussed below, the wide-ranging nature of these exemptions could be used to circumvent regulations by well-resourced corporate actors. At the time of writing, the negotiations between the institutions on the agreement were ongoing. On 15 June 2017, the Conference of Presidents of the EP (consisting of the President of the EP and political group leaders within the EP) approved the Parliament’s negotiating mandate and on 6 September 2017 a tripartite meeting between the Commission, the EP and the Council took place ahead of the formal inter-institutional negotiations on the agreement.

In the interim, the Commission announced in November 2014 that from 1 December that year it would publish details of all meetings of Commissioners, their Cabinet Members and Commission Directors General with all interest representatives, indicating that such meetings should, as a default, be limited to individuals and organisations registered on the transparency database [27]. However, the declaration stopped short of a complete ban on meetings with non-registered actors and left open the possibility of meetings with lower-level officials. In December 2016, MEPs voted to adopt measures limiting meetings to registered lobbyists, but this was widely interpreted as non-binding and has not been applied by all members. The Juncker transparency agenda was frequently cited by respondents in relation to Article 5.3. Below, we report our findings on explicit measures undertaken to implement Article 5.3, beginning with an analysis of relevant documents.

## Results: Implementation of Article 5.3 in the EU

Commission reports to the FCTC Secretariat, covering the years 2007, 2010, 2012, 2014 and 2016, focus almost entirely on generic transparency and ethical behaviour guidelines [31–35]. The reports for all years answer the yes/no question on whether measures have been taken to implement Article 5.3 in the affirmative, with most years making reference simply to the *Staff Regulations* for EU officials (set out in Regulation No 31 [EEC], 11 [EAEC]). The latter are reported to provide “legally binding ethical standards” on officials that ensure the application of “principles of independence, impartiality, objectivity and loyalty” [36]. In response to a question on the use of the WHO Guidelines on Article 5.3, the 2014 report to the FCTC Secretariat adds that the Commission “consults stakeholders to the extent necessary for the elaboration of the appropriate regulation on tobacco products” and that the DG for health “publishes the minutes of meetings with industry on its website”. The report for 2016 elaborates on this by making reference to additional “ethical and integrity rules”, notably the *European Code of Good Administrative Behaviour* [37], the *Code of Conduct for Commissioners* [38], and the *Practical Guide to Staff Ethics and Conduct* [39]. No reference is made to the EP’s *Code of Conduct for Members of the European Parliament with Respect to Financial Interests and Conflicts of Interest* (see below) [15]. The reports to the FCTC Secretariat further mention rules on transparency, notably Regulation 1049/2001 on public access to documents [40] and the Transparency Register, noting that “as of 1^st^ December 2014 Commissioners, their Cabinet members and Directors General publish information on the meetings they hold with lobbyists” [35]. Only in the 2016 report is the question on “ensuring that the public has access, in accordance with Article 12(c), to a wide range of information on tobacco industry activities” answered in the affirmative. Since the *Staff Regulations* and the *European Code of Good Administrative Behaviour* relate to all EU officials, we discuss them here. Other documents and practices cited in reports, which relate specifically to the Commission and/or the EP are discussed in the relevant sections below.

The *Staff Regulations* govern relations between EU institutions and their officials. Title II, Article 11, of the Regulations contains provisions that deal with potential conflicts of interest, requiring officials to refuse “any honour, decoration, favour, gift or payment” (without expressed permission) and to disclose any actual or potential conflict of interest when being considered for appointment. Such general measures go some way to meet the WHO’s Article 5.3 guideline recommendations 4.1, 4.2, 4.5 and 4.10 on conflicts of interest, but they are not specific to the tobacco industry. This lack of specificity is important, since recommendation 4.2 specifically states that Parties should “formulate, adopt and implement a code of conduct for public officials, prescribing the standards with which they should comply in their dealings *with the tobacco industry*” (emphasis added). Furthermore, the recommendations indicate that Parties should “inform and educate all branches of government and the public about […] the strategies and tactics used by the tobacco industry to interfere with the setting and implementation of public health policies” (1.1) and the industry’s practice of using third parties to further their interests (1.2). Such measures require a specific policy and code of conduct relating to the tobacco industry, in addition to generic policies on conflicts of interest and transparency. Title II, Article 16, of the *Staff Regulations* contain provisions which meet WHO recommendation 4.4. These require officials to inform their institution if they intend to engage in any occupational activity within two years of leaving service; if the activity could lead to a conflict of interest, the official could be forbidden from taking it up or certain conditions may be attached to their acceptance. Senior staff are prohibited from lobbying their former institution, on matters for which they were responsible, for a period of 12 months after leaving office.

The *European Code of Good Administrative Behaviour* sets out general principles of good administration which apply to relations between the EU institutions and the public, but does not directly address the measures specified in the WHO’s Article 5.3 guidelines. However, the same document contains five public service principles, two of which – “integrity” and “transparency” – are of direct relevance to Article 5.3, although they do not deal specifically with the tobacco industry. The “integrity” principle includes the following statement, which relates to WHO recommendations 4.1, 4.6, and 4.10 on avoiding conflicts of interest:

Civil servants should not place themselves under any financial or other obligation that might influence them in the performance of their functions, including by the receipt of gifts. They should promptly declare any private interests relating to their functions. Civil servants should take steps to avoid conflicts of interest and the appearance of such conflicts.

The transparency principle, which is relevant to WHO recommendation 2.2, specifies that public servants should “keep proper records and welcome public scrutiny of their conduct”, a statement that is relevant to the European Ombudsman’s inquiry into the Commission’s implementation of Article 5.3, discussed below.

### The Commission

The central role played by the Commission within the EU’s institutional architecture, suggests that it would be the most likely of the institutions to have fully-developed policies to manage engagement with the tobacco industry. Of the documents cited in the Commission’s reports to the FCTC Secretariat, the *Code of Conduct for Commissioners* (hereafter CCC) [38] deals specifically with the responsibilities of Commissioners. This contains a number of provisions that go some way to meet WHO’s recommendations on the avoidance of conflicts of interest (recommendations 4.1–4.11). WHO recommendations 4.1 and 4.2 advise Parties to “mandate a policy on the disclosure and management of conflicts of interest” (4.1) and adopt a code of conduct (4.2). These recommendations are met by the CCC in a general sense, although not specifically in relation to the tobacco industry. WHO recommendation 4.4 advises Parties to adopt a policy requiring officials to “inform their institutions about any intention to engage in an occupational activity within the tobacco industry, whether gainful or not, within a specified period of time after leaving service.” While not relating specifically to tobacco interests, and in a similar vein to the EU *Staff Regulations*, the CCC requires former Commissioners to inform the Commission of any intent to engage in an occupation during the 18 months after they have ceased to hold office, a provision that will be increased to two years from February 2018 once a new CCC enters into force [41]. Where such an occupation is related to the content of the portfolio of the Commissioner, the opinion of an “Ad Hoc Ethical Committee” is sought and, during that 18 month/two year period, former Commissioners may not lobby Commission staff on matters for which they have been responsible.

WHO recommendation 4.6 recommends that Parties should require officials to “declare and divest” themselves of tobacco industry interests. The CCC requires Commissioners to declare any financial interest that might create a conflict of interest. Under the new code from February 2018 they will be required to declare investments above €10,000 regardless of whether there could be a conflict of interest and, in cases of a conflict of interest arising from a financial interest, the President of the Commission will be able to request divestiture or placement in a blind trust. WHO recommendation 4.10 advises Parties to prohibit officials from accepting payments, gifts or in-kind services from the tobacco industry. The CCC prohibits Commissioners from accepting any gift with a value of more than €150 and from accepting hospitality, “except when in accordance with diplomatic and courtesy usage” [41: 7]. While the measures in the CCC therefore go some way to meeting some of the WHO recommendations on conflict of interest, they do not deal specifically with tobacco, and do not address some recommendations in this area at all. They also relate only to the 28 Commissioners and do not cover influential officials below the College.

Commission officials below the rank of Commissioner are covered by the *Staff Regulations* and the *Practical Guide to Staff Ethics and Conduct*. The *Practical Guide* is not legally binding but rather sets out in clear fashion the requirements of the *Staff Regulations* and emphasises the need for officials to behave in an ethical manner. In relation to interest groups, it states that the Commission “has an obligation to listen to all parties” but notes that, where meetings with interest group representatives are held, “[a] written record of such meetings should be ensured where these contain important information or may involve action by the Commission. Such reports should be registered and filed” [39: 12].

However, the decision of the EU Ombudsman, Emily O’Reilly, in an inquiry into the Commission’s implementation of Article 5.3 was extremely critical, particularly in relation to transparency measures (WHO recommendations 2.2 and 5.1–5.5) [42]. The Ombudsman’s inquiry followed a complaint in January 2013 by Brussels-based NGO Corporate Europe Observatory (CEO) and four other non-governmental organisations (NGOs) that a number of Commission officials had failed to disclose meetings with tobacco industry representatives and that – with the exception of the DG for Health, DG Sanco (now DG Santé) – the Commission had failed to guard itself adequately against tobacco industry influence. In its opinion on the case, the Commission insisted that its generic transparency guidelines were consistent with Article 5.3 and argued that, although Article 5.3 itself was legally binding, the WHO implementation guidelines were not [43]. While DG Sanco proactively publishes the minutes of all meetings with tobacco industry representatives, the Commission argued that other DGs need not do so because Regulation 1049/2001, which provides for public requests to access documents, was sufficient to ensure transparency. Finally, the Commission argued that its Legal Services were not covered by the obligations of Article 5.3 when meeting with legal representatives of the tobacco industry.

Interview data indicate that levels of awareness of Article 5.3 and responses to it varied between DGs, and suggest that restricting proactive transparency measures to DG Sanco creates ample opportunities for tobacco companies to bypass it. As one official with internal knowledge of Article 5.3 compliance within the EU institutions commented:

They basically were not publishing proactively their meetings. They weren’t publishing their minutes, only DG health do. DG health do do this. But of course the tobacco industry bypassed DG Health. They totally ignored DG Health really. Well they don’t ignore it but they lobby the legal services, they lobby the cabinets, they lobby all the DGs across issues. And the Director as well. So the Commission says that DG Health is implementing it and that’s enough. We will say that clearly it’s not enough because policy in the Commission is made by the College. Of course, the legal services have a huge say; the Cabinets, other DG’s, you know, industry DG or whatever.

This was mirrored by a respondent from CEO, who commented:

So the Commission – most of any EU institutions – would be the first to take these rules very seriously and that clearly has not been the case. DG Sanco started very late with introducing this online transparency around meetings with the tobacco industry or meetings on tobacco legislation in 2011. From 2011, I think, they started uploading these – the list of meetings and minutes from meetings, which is a good thing – but it amazingly doesn’t seem to be written down anywhere what the rules are. It’s just a practice that was introduced in DG Sanco but there is no paper trail.

The lack of a formal policy document codifying procedures is indicative of the piecemeal and informal approach to Article 5.3 compliance. Moreover, the historical lists of meetings appear to be incomplete, omitting certain meetings with the industry [44]. Despite its shortcomings, DG Sanco’s approach compared favourably to practices within other DGs, in which there was no online reporting requirement or other measures in place beyond the Commission’s general transparency and ethics rules. The same respondent thought the lack of specific compliance structures in place in relation to Article 5.3 reflected the low level of seriousness with which the matter was treated by the Commission:

And when we, together with other NGOs, challenged the Commission on [Article 5.3 compliance], and we’ve had this long exchange of letters in which the Commission would simply repeat its statement but without making any attempt to be convincing or ... So there must have been an internal decision that this was not…. not only it was not important; it was important *not* to implement the rules.

The respondent speculated that this obstructionism reflected the overtly pro-business orientation of the previous Commission under the Presidency of José Manuel Barroso, and concerns the Commission had about the consequences of Article 5.3 implementation for their relationship with business:

people in the Barroso Commission considered any move towards implementing Article 5.3 as being a dangerous precedent that could lead to a more comprehensive transparency and ethics, demands on the Commission also for other areas and, therefore, they really dug their heels in the sand and refused to do anything.

The same respondent suggested that difficulties in restricting meetings with tobacco industry actors reflects also the culture of the Commission and the sense of obligation which officials felt to meet with industry stakeholders.

Other respondents argued that the Commission’s better regulation agenda had been used as a pretext for not implementing restrictions on tobacco industry access to policy makers, given the apparent need for dialogue with industry in order to reduce regulatory burdens. As one EU official commented:

you’d have some lawyer saying, “Oh well, under better regulation we’d have to talk to everybody about it.” So you hear a lot of the similar arguments, let’s say, coming from people which actually came originally from the tobacco industry. But it’s difficult […], in general health is never the top priority politically.

The same official stated that, for many Commission officials, the WHO’s Article 5.3 guidelines are seen as optional:

Now they would’ve said, “Oh, the guidelines are not legally binding, blah, blah, blah,” but doesn’t mean you can’t do it. You know, it’s still a choice to do it.

A related point concerned definitional issues of what constitutes a tobacco industry actor, and thus who is covered by the restrictions on policy access and influence set out in Article 5.3. These issues arise perhaps most obviously in relation to trade associations and cross-sectoral business organisations – such as Business Europe and the American Chamber of Commerce (AMCHAM) as well as national associations such as the Federation of German Industry (BDI) and the Dutch Employers Federation (VNO-NCW) – which have tobacco industry members, whose interests they represent and which may include TTC personnel in delegations attending meetings with officials. As an NGO actor with knowledge of tobacco industry lobbying of the Commission in relation to the TTIP negotiations underway during the period of analysis commented:

I know [tobacco companies are] working through AMCHAM […] on this issue. They are certainly members in a number of AMCHAM committees where TTIP is discussed. They have certainly contributed to AMCHAM fact sheets and briefings and whatnot, so there’s a lot of use of the trade associations. But no, we have not invested a lot of time into actually documenting all of these things.

The Juncker transparency agenda, and the mandatory register for lobbyists, was frequently cited by respondents in relation to Article 5.3. However, interviewees were sceptical about the degree of change they would bring about. First, as with the issues in defining the tobacco industry, there was some ambiguity about precisely which actors count as lobbyists, and, therefore, must be included in the register. For the purposes of Article 5.3 monitoring, a law firm representing multiple clients (often undisclosed due to privilege rules), for example, may meet with Commission officials, but it will be unclear from this if they discussed issues relating to tobacco clients. Discrepancies in the information provided by the Commission to MEPs and the Ombudsman’s enquiry, relating to meetings between the Commission Legal Services and lawyers acting on behalf of the tobacco industry, highlight considerable uncertainty amongst Commission officials about whether meetings with such third party actors constitute engagement with the tobacco industry and thus fall under the remit of Article 5.3. Whilst officials had disclosed meetings with tobacco company representatives to MEPs, they had omitted them from evidence to the Ombudsman’s enquiry on the grounds that “the Legal Service do not, in general, regard contacts with lawyers representing law firms providing legal advice to companies, as being contacts with the industry itself” [45]. This raises questions about whether tobacco clients need to be explicitly referenced in meetings with lawyers for Article 5.3 to be relevant, or whether indirect representation – for example, in relation to issues of common concern to the tobacco industry and other industries – is enough for a law firm with tobacco industry clients to be considered as a tobacco industry actor for the purposes of Article 5.3. These issues remain unclear, even in the WHO guidelines.

Second, while the occurrence of meetings must be declared, their content, and even precise details of who attended, is not minuted to allow scrutiny of the issues discussed. As one respondent with internal knowledge of the EU institutions commented, the fact that meetings with officials are recorded in this way is highly problematic in terms of Article 5.3 implementation:

So say you meet Business Europe. What does Business Europe of course do? You can bring in your Philip Morris person and you can bring in anybody. You don’t know. So it should always say who is lobbying from the lobby side so then you exactly know, is it the CEO [Chief Executive Officer], is it the lawyer, is it the trade guy? So the names of the lobbyists […] the names should be published of who comes because often the lobbyists have different hats, under different guises.

Third, as discussed above, the disclosure rules are limited to the most senior officials, leaving open the possibility for more junior, yet nonetheless highly influential functionaries (for example at the level of Deputy Director General, Heads of Unit and others), to meet lobbyists and industry actors without the need for disclosure. This offers a straightforward route to circumnavigate even these rudimentary measures. Fourth, meetings are only one mechanism among many (increasingly informal) channels which lobbyists may use to engage and potentially influence policy makers. These forms of engagement would fall outside the remit of the transparency register and disclosure rules and are very hard to capture, posing further issues for Article 5.3 monitoring and compliance. As the same respondent commented:

The Junker Commission do proactively publish meetings at the high level, so this is a very good thing. But first of all, 95% of Commission officials are not covered. But also, influence happens in many, many, many ways. Not just meetings. Meetings are probably the most basic and – you know, you’re never going to regulate all human activity. It’s just never going to happen. But phone calls, dinners, you know, social evenings. We see more and more in Brussels – it tends towards social events because you’re not captured then by the transparency register. It’s not a meeting as such. If the organization doesn’t have policy positions then it’s a social gathering you don’t have to register. But you’re still influencing. You’re still making personal contacts.

While it is possible to request details of meetings held by other officials via freedom of information (FOI) rules codified under Regulation 1049/2001, this is an arduous and time-consuming process for organisations, such as NGOs, with limited resources. Access to documents can often be denied. As attempts by organisations such as CEO to use FOI requests in the context of the TTIP negotiations reveal, even where documents are released, the information disclosed in them is often heavily redacted on the grounds of data protection, rendering it unusable [46]. Even for those within the EU institutions it was difficult to obtain information about Commission contacts with the tobacco industry during the TTIP process. An NGO representative cited delayed and reluctant compliance with FOI requests as an example of this:

And then it took several back and forth, so the Commission didn’t respond in the prescribed timeframe which is 15 working days. They prolonged the deadline. Then after the prolonged deadline, they still didn’t respond. And then at one point, they did release, I think in the first response, they released two documents: one meeting request and then a report of that meeting from JTI [Japan Tobacco International]. But in the correspondence before, they had mentioned other documents regarding Philip Morris and also BAT, which weren’t included in the response, which is why, then, [someone within the EU institutions] filed a complaint, which you can do based on the regulation. […] I think he did receive all those documents. I’m not sure. But at least some more but they were nearly 100% censored. So you can see, “A-ha, BAT wrote a letter to the Commission.” It’s 5–6 pages long. They outline apparently many concerns that they have or interests, with regards to the EU trade negotiations, but all of this is blackened [i.e. redacted].

The respondent explained the reasons given for redaction were that the documents potentially contained commercially sensitive information, that it would endanger international relations or interfere with internal decision making processes. Article 9 of Regulation 1049/2001 sets out the grounds on which the release of documents originating in the EU institutions or member states marked “top secret,” “secret” or “confidential” may be refused “in accordance with the rules of the institution concerned, which protect essential interests of the European Union or of one or more of its member states in the areas covered by Article 4(1)(a), notably public security, defence and military matters.” As is evident from heavily redacted documents related to tobacco industry contacts previously released and discussed above, this provision offers wide scope to the Commission to withhold material of relevance not only to Article 5.3 compliance but to the wider transparency initiatives often cited by the Commission in this context.

### The European Parliament

Compliance with Article 5.3 within the EP was even less substantial than in the Commission. In terms of documents analysed, no references are made to the EP or its policies in Commission reports – made on behalf of the entire EU – to the FCTC Secretariat. Despite this, relevant policies are in place, and associated documents were identified. The EP introduced its *Code of Conduct for Members of the European Parliament with Respect to Financial Interests and Conflicts of Interest* in 2012 [15]. This requires MEPs to disclose any financial benefits received within 30 days via an established disclosure mechanism, including their occupation(s) during the three-year period before taking up office in the Parliament. It does not impose any restrictions on MEPs’ subsequent employment, other than to prevent them from benefiting from “facilities granted to former Members” should they engage in professional lobbying. An Advisory Panel oversees the functioning and enforcement of the code, advising MEPs on compliance and producing an annual report on its activities. In addition, officials within the EP are covered by a separate *Guide to the Obligations of Officials and Other Servants of the European Parliament - Code of Conduct* [16]. In this section, we confine ourselves to discussing Article 5.3 implementation among MEPs. The omission of references to the MEPs own code of conduct within the Commission’s reporting demonstrates not just important shortcomings in Article 5.3 compliance, but the Commission’s focus on officials – and principally those within the Commission – to the exclusion of elected representatives at the EU level. It reflects also the different roles of the institutions, and the different traditions of transparency, access and conflict of interest (COI) that exist within the Commission and the EP, and in relation to legislatures and bureaucracies more generally.

The lack of EP-specific compliance mechanisms was evident also among interviewees. Asked if there were measures in place to ensure compliance with the FCTC, one MEP answered bluntly: "no." A respondent from CEO concurred, stating "in the European Parliament indeed there is nothing […] there really was no form of implementation." In addition, interviews with MEPs revealed a widespread lack of knowledge of the FCTC, and of Article 5.3 in particular, even amongst those active in the area of health. This was particularly the case prior to the enactment of the TPD. Asked if they were aware of the FCTC before this point, one MEP involved in the deliberations on the TPD commented:

No, in fact, the first time I realized, and I’m quite embarrassed about that, but the first time I realized that we had this convention was when [another MEP] mentioned it during the first exchange of views on the Tobacco Directive. […] in ENVI, showing up saying that they wanted to follow that and they recommended all the colleagues in the department to follow that. And then I [thought] “Oh, my God.” And then I took it up and read it and I talked with, for instance, the [cancer NGOs in my home country] about it as well, asking them about what is their point of view on this and how should it be interpreted etcetera. So, it gave me something to think about.

However, the volume of industry lobbying which occurred during the TPD process [8], including the ‘Dalligate’ affair, allied to the efforts of MEPs with an understanding of the issue to explain the significance of Article 5.3 to colleagues, led to increased awareness. A respondent from an NGO engaged in tobacco-control issues suggested that this, in turn, created the impetus for the introduction of more substantial compliance mechanisms:

I guess during the Tobacco Products Directive there was no way they had time to think about [Article 5.3], although they really did feel the pressure. And I suppose this has created the momentum maybe for some of them who have stayed […] to actually do something about it. Whether they’re successful or not, and what would be the mechanisms, I don’t know.

Following the conclusion of the TPD, MEP Gilles Pargneaux led the formation of an ‘anti-tobacco’ group within the EP with the specific objective of addressing tobacco industry lobbying and influence over policy making at the European level. The fallout from the TPD process also set in motion attempts to address the issue of tobacco industry lobbying and Article 5.3 compliance across the institution. As the NGO respondent continued:

At the moment, the Conference of Presidents [of the EP] – so including [EP President Martin] Schultz – are discussing 5.3 in the parliament. And they’ve asked for a legal appraisal, but it was on their agenda to discuss and they look as if they don’t want to do much, even though the Socialists […] were not necessarily against; the Greens are not necessarily against. There are possibilities to do something with the Parliament. At the moment there’s nothing. It is clear that it would have to be enshrined in the rules and procedures of the Parliament – yeah, we are working on that. But at the moment there is nothing.

Despite the extensive lobbying that occurred during the TPD, opposition to restrictions on engagement with tobacco industry actors was evident amongst some MEPs. Whilst many mainly Green and S&D MEPs supported moves to address Article 5.3, opposition to this was both widespread and evident at the highest levels of the EP, apparently including EP President, Martin Schulz. As one NGO respondent highlighted:

There were at least two letters sent to Martin Schulz from MEPs asking for [measures to comply with Article 5.3] and that was brushed under the carpet by Mr. Schulz, who is not somebody who has too much of an appetite for codifying ethics rules or transparency rules or anything of that kind, it’s not important for him. […] So I think that’s very clear, there’s been no institutional … the institution has not taken responsibility at all. There have been initiatives by individual MEPs and by the Green Group to unilaterally report on meetings they have had with the tobacco industry and also, I guess, some more cautious approach, but that’s all.

The opposition of the EP President to such proposals is in keeping with the generally weak enforcement of Article 5.3 within the Parliament.

As in the Commission, one of the principal ways of addressing concerns about tobacco industry influence in the EP appeared to be through attempts to increase transparency in the conduct of business and interactions with the tobacco industry. However, the rhetorical commitment to openness appears to have been difficult to implement in practise, with TTCs often able to avoid scrutiny of their activities in subtle ways. For example, as the previous respondent continued:

I remember that there have been meetings also connected to the TPD. Open meetings where on the one hand it’s so-called transparent and open and they have tobacco representatives there but as soon as the meeting starts they say, “This is a meeting with Chatham House rules.” Which is a really obvious trick to openly – so-called openly, to lobby MEPs and assistants to give credibility to tobacco industry arguments. At the same time, nothing can go out.

Citing transparency measures as sufficient in the context of tobacco industry access and Article 5.3 compliance is in keeping with the findings above in relation to the Commission and EU-wide compliance reporting to the FCTC Secretariat.

However, the implementation of Article 5.3 in the EP presents additional challenges beyond those encountered in relation to the Commission. These arise from the specific competencies assigned to the EP within the treaties, the complexities of its working practices and the issues which derive from the presence of differing (and at times conflicting) democratic and political cultures from different member states within a single institution, in addition to the ideological divisions between party groups common to all parliaments. As with the Commission, there were definitional issues around what constituted contact with the tobacco industry when they acted via third parties, especially those that would not be considered obvious industry allies. Whilst the connections between trade associations and tobacco company members are perhaps unsurprising, other organisations such as trade unions were also used as conduits for tobacco industry interest representation, particularly for politicians with close connections to the labour movement who may be less inclined to meet with business groups. As one S&D Group MEP stated:

what struck me more is I was contacted by trade unions. In general, the big one, the umbrella organization in [my country], but also some concrete organizations representing the tobacco workers. We had a very big tobacco production in [my country] years ago […] and we had a quite strong trade union working with people working in the tobacco industry and the food industry etcetera. And they were quite active but I have a very good relationship with them and they know my position and they respect it and that’s how it is.

The role of trade unions in this context creates a challenge for Article 5.3 implementation, and for the wider transparency agenda, in determining which entities should be considered civil society bodies and/or social partners and thus be excluded from the groups required to register as lobbyists.

Two further barriers to the effective implementation of Article 5.3 within the EP emerged from interviews. First, it was evident that there were very different conceptions of COI which informed the position of MEPS from different member states on whether they were able (or even obliged) to meet with stakeholders from different sectors, particularly where they had some interest in the MEP’s region itself. Whilst Commissioners and officials must (in principle at least) act on behalf of the Union as a whole, rather than their home states, MEPs are explicitly mandated to represent their constituents and to maintain close links with the countries and regions which elected them. In addition, specific conceptions of MEPs’ independence of mandate led to a reluctance to preclude meetings with any specific group of actors, even the tobacco industry. This approach assumes that the underlying logic of Article 5.3 is derived from a specifically Anglo-Saxon conception of COI, which was alien to some other traditions of parliamentary representation within member states. The different status of MEPs and officials is recognised formally in documents establishing the EU’s current transparency regime. The inter-institutional agreement establishing the current voluntary registration mechanisms for lobbyists states explicitly that “the register shall respect the rights of Members of the European Parliament to exercise their parliamentary mandate without restriction” [24].

It is noteworthy how widespread the belief was that the EP could not be subject to similar forms of regulation to the Commission in order to implement Article 5.3. Even NGO respondents strongly in favour of tobacco control seemed to accept that parliamentarians were different from functionaries in terms of the mechanisms which could be put in place for the purposes of Article 5.3 implementation, given the different role they performed, and their mandate to represent wider society. As one NGO representative commented:

I don’t know many countries where the MPs have to record all the meetings of the industry. Because as a legislator, you should have a different [role]. It’s very difficult to convince MPs to accept 5.3. But outside DG Sanco, that’s not normal.

Second, it was argued by some respondents that the EP lacked an adequate legal basis in the EU treaties to implement a mandatory code of conduct for MEPs, which could preclude or at least regulate engagement with the tobacco industry in line with Article 5.3. One respondent from a health NGO claimed that such regulations would in fact require a change to the treaties, and that any such change would be politically problematic, despite the obligations included in the FCTC. In this context, they argued that a more effective approach than mandatory codes of practice and disclosure requirements for engagement with tobacco industry actors may be for ‘soft law’ approaches in which MEPs voluntarily disclose their meetings. In addition to the inherent weaknesses of self-regulatory approaches, a voluntary disclosure requirement would also fall some way short of the types of safeguards against industry influence foreseen by WHO guidelines on Article 5.3 compliance.

One of the issues appeared to be that the legal obligations contained within the FCTC were not taken seriously by key political actors and some individual MEPs. As one MEP commented:

And I think it was only after the Philip Morris thing [i.e. a leak of internal company documents (see [8]) revealing the company’s lobbying strategy] that they started to understand that this did matter. I think it was seen as “oh, god, you know, what is this” and – again, a bit over the top. But I mean, I kept trying to point out our government sort of signed up to this, EU signed up to this framework convention and we need to take it seriously, it’s not some joke.

This attitude to the EU’s obligations under international law evident in relation to the FCTC and Article 5.3 compliance stands in clear contrast to the approach taken to EU law. The perceived need for an explicit EU treaty basis for Article 5.3 compliance measures amongst MEPs, outlined above, demonstrates the primacy accorded to EU law over other international treaty obligations.

## Discussion

As with Fooks et al.’s [3] study of national implementation of Article 5.3, we found that much compliance activity at the EU level is passive or indirect; i.e. resulting from existing policies, processes, mechanisms, and codes of conduct, which are generic in nature and not directed specifically at achieving the objectives of Article 5.3. Most such policies in the European Commission and the EP focus only on general guidelines concerning transparency, conflicts of interest and ethical behaviour. However, this approach does not achieve the objective of full compliance with either the letter or spirit of Article 5.3. For example, the WHO’s Article 5.3 implementation guideline 4.2 recommends that parties adopt a specific code of conduct relating to the tobacco industry. The measures put in place by the Commission and the EP appear inadequate for the purpose of providing full transparency even in cases of non-tobacco business lobbying, and are certainly insufficient to meet the specific requirements of Article 5.3.

On transparency, the WHO’s Article 5.3 guidelines recommend that Parties should limit interactions with the industry to those that are strictly required for its effective regulation (2.1) and that, where such interactions are necessary, they should be conducted transparently (2.2). While DG Sanco (now Santé) has implemented a working norm requiring officials to publicly record meetings and minutes, this remains insufficiently formalised and codified, and even such basic measures have not been extended to other DGs. Furthermore, the practice of Commissioners, members of their Cabinets and Directors General to meet only with organisations that are on the Transparency Register and to publish details of such meetings does not extend to more junior officials. As Fooks et al. [3] found, these kinds of selective and incomplete measures offer extensive opportunities for tobacco companies to shift lobbying activities to other venues, i.e. to target officials or DGs not covered by these provisions; a strategy that was pursued extensively by TTCs in their lobbying over the TPD [8]. Moreover, practices based on working norms rather than clear, written guidelines, as DG Sanco’s practices appear to be, are vulnerable to poor implementation or to lapsing when current post-holders move on [3].

In her decision on the inquiry into the Commission’s implementation of Article 5.3, the Ombudsman found that the Commission’s consistent refusal to proactively apply the transparency policy of DG Sanco across all DGs constituted maladministration [42]. While Regulation 1049/2001 [40] provides for some retrospective level of transparency, it places the burden on individual actors to identify and seek out relevant material, rather than on the Commission to place this in the public domain as a matter of course. Thus, unless a citizen makes a request for access to documents, or an MEP puts a relevant question to the Commission, such meetings would remain undisclosed [45: paragraph 35–36]. Furthermore, Regulation 1049/2001 could not be effective where an official had made no official record of a meeting. How can actors request documents to a meeting they do not know has occurred? The Ombudsman noted that Article 5.3 requires FCTC Parties to *act* – i.e. to take proactive measures – to protect health policies from the tobacco industry, and thus restricting the disclosure requirement to DG Sanco alone left the industry able to access other DGs in a non-transparent way. She recommended that the more robust transparency measures put in place by DG Sanco be adopted across the Commission, including its Legal Services. She further noted the point discussed above, that the Commission’s policy – to publish information on meetings held by Commissioners, their Cabinets and Directors General with outside organisations and to limit those meetings to organisations on the Transparency Register – covered only a limited number of top officials. In a strongly worded comment, the Ombudsman stated that she would “have expected that the experience gained by the Commission from the adoption of the TPD, widely acknowledged as the most lobbied dossier in the history of the EU institutions, would have convinced it of the need to strengthen further its ethical rules” [42: paragraph 23].

Aside from its limited transparency measures, the Commission also referred in its response to the Ombudsman to generic guidelines for its staff requiring ethical behaviour, such as the EU *Staff Regulations* and the CCC. Such guidelines meet the requirements of the WHO Article 5.3 guidelines on the avoidance of conflicts of interest (4.1–4.11) to some extent, but they do not deal specifically with tobacco industry interests, and do not cover all of the WHO’s COI recommendations.

As noted above, the WHO’s Article 5.3 implementation guidelines [2] go beyond simple transparency and COI measures, to include measures on awareness raising and limiting interactions with the industry. WHO guidelines on awareness raising stipulate that Parties should “inform and educate all branches of government and the public about… the need to protect public health policies for tobacco control from commercial and other vested interests of the tobacco industry” (1.1) and that they should “raise awareness about the tobacco industry’s practice of using individuals, front groups and affiliated organizations to act, openly or covertly, on their behalf” (1.2). Our interviews reveal an extensive lack of awareness of the requirements of Article 5.3, especially among MEPs, with even those directly involved in tobacco-control issues unaware of Article 5.3 before their engagement with this topic. We found no evidence of proactive attempts by EU institutions to inform policy makers and officials about Article 5.3 or to educate them about the political strategies of tobacco companies, other than the ad-hoc actions taken by those working on the passage of the TPD. Without a specific policy and code of conduct implementing Article 5.3 it is extremely difficult to meet these awareness-raising requirements, or to effectively monitor tobacco industry political activity and fully meet the requirements of guideline recommendations 5.1–5.5, which specify the provision of information by the industry and its proxies.

While intensive industry lobbying over the TPD has raised awareness of Article 5.3 within the EP to some degree, this has been a slow and gradual process and there remains resistance to implementation among some MEPs that are aware of it. This appears to be exacerbated by differences in political culture between member states about how conflicts of interest should be handled. It also raises issues about the legal mandate that MEPs take from the EU treaties and whether this allows for a requirement to limit interactions with stakeholders. Whilst we must leave it to legal scholars to evaluate the veracity of these claims, it is sufficient to note here the continued opposition to increased transparency and reporting requirements and the rhetorical and political use of such arguments to this end. Our data suggest that some MEPs see a strong mandate for independence in the treaties, and that they take the legal obligations imposed on the EU by the FCTC as an international treaty less seriously than those contained in the EU’s own foundational treaties. As elected representatives, accountable ultimately to their constituents, MEPs represent a distinct category of actor to appointed functionaries, with different implications and challenges in terms of Article 5.3 compliance. However, Article 5.3 does not prohibit all meetings with industry representatives; it simply requires that these be kept to those that are strictly necessary and that where they take place they should be transparent [47]. We note also that Transparency International recommends that the EP routinely publish “legislative footprints”, i.e. track and publish information on contacts and inputs received during the process of creating policies and laws [48]. The adoption of such transparency measures was further recommended in a 2008 resolution of the EP, and a small number of MEPs have already voluntarily adopted these practices [48]. A mandatory code of this kind would in no way restrict MEPs’ freedom of action; rather it would simply increase transparency and, therefore, the accountability of representatives to their constituents.

The Commission’s claim that existing transparency and ethical behaviour guidelines were adequate to achieve Article 5.3 compliance, and that tobacco industry lobbying was now less of a concern since the adoption of the TPD, reveals a clear failure to understand the nature of industry political strategies and the need to guard against these [42]. It fails to understand the ongoing, and often indirect, nature of tobacco industry political activity, which targets not only explicit tobacco-control policies, but also other policy areas, such as trade negotiations, that may offer the industry opportunities to advance their interests [17, 49]. As the Ombudsman noted, the Commission’s approach “reflects a very short term and random approach at the expense of a comprehensive and legally sound framework” [42].

Similarly, the EU institutions appear to have failed to understand, and to make officials aware of, the use of third parties (such as cross-industry trade associations, consultancies, law firms and even trade unions) by tobacco companies (as recommended by Article 5.3 guideline 1.2). Our interviews revealed extensive use of third-party lobbying over the TPD and TTIP negotiations, including by law firms acting on behalf of the industry [see also 8, 45]. Yet the Commission, in its response to the Ombudsman, was unwilling even to accept that its Legal Services should fall under the provisions of Article 5.3. The scope for third-party bodies to lobby on behalf of tobacco interests appears to be extensive in the EU, with little political will evident to guard against this. We found only limited evidence of an attempt to implement the WHO Article 5.3 guidelines on limiting interaction with industry representatives themselves (recommendation 2.1), let alone third parties representing their interests. Our findings are presented in summary form in Table 2.

**Table 2:** WHO Guidelines for Implementation of Article 5.3 of the FCTC (abridged^a^): European Commission and European Parliament Implementation

General Recommendations	Specific Recommendations	Commission	Parliament ^b^
Raise awareness about the addictive and harmful nature of tobacco products and about tobacco industry interference with Parties’ tobacco control policies.	1.1 Parties should inform and educate all branches of government and the public about the addictive and harmful nature of tobacco products, the need to protect public health policies for tobacco control from commercial and other vested interests of the tobacco industry and the strategies and tactics used by the tobacco industry to interfere with the setting and implementation of public health policies with respect to tobacco control.	Not met. Little evidence of awareness raising among officials about the need to protect policies from industry interference, especially outside of DG Sanco (DG Santé).	Not met. Very low awareness among MEPs of the need to protect public policies from industry interference.
	1.2 Parties should, in addition, raise awareness about the tobacco industry’s practice of using individuals, front groups and affiliated organizations to act, openly or covertly, on their behalf or to take action to further the interests of the tobacco industry.	Not met. No evidence of awareness raising about the use of third parties by the industry.	Not met. No evidence of awareness raising about the use of third parties by the industry.
Establish measures to limit interactions with the tobacco industry and ensure the transparency of those interactions that occur.	2.1 Parties should interact with the tobacco industry only when and to the extent strictly necessary to enable them to effectively regulate the tobacco industry and tobacco products.	Not met. Officials do not uniformly limit their interactions with the industry.	Not met. MEPs do not uniformly limit their interactions with the industry.
	2.2 Where interactions with the tobacco industry are necessary, Parties should ensure that such interactions are conducted transparently. Whenever possible, interactions should be conducted in public, for example through public hearings, public notice of interactions, and disclosure of records of such interactions to the public.	Partially met. DG Sanco publishes minutes of meetings that do take place with the industry, but other DGs do not. Commissioners, members of their Cabinets and Directors General meet only with organisations that are on the Transparency Register and publish details of such meetings, but this does not extend to more junior officials. Regulation 1049/2001 allows access to some information by the public, but places the burden on the public rather than the Commission and may provide partial (redacted) information.	Not met. MEPs do not uniformly record their interactions with the industry. The current voluntary Transparency Register explicitly recognizes MEPs’ freedom of mandate and the associated right to meet third parties. Regulation 1049/2001 allows access to some information by the public, but places the burden on the public rather than the institution and may provide partial (redacted) information.
Avoid conflicts of interest for government officials and employees.	4.1 Parties should mandate a policy on the disclosure and management of conflicts of interest that applies to all persons involved in setting and implementing public health policies with respect to tobacco control, including government officials, employees, consultants and contractors.	Met by general regulations and guidelines, including the *Staff Regulations* and the *Code of Conduct for Commissioners*, although these are not specific to tobacco.	Met in part by the *Code of Conduct for MEPs with Respect to Financial Interest and Conflicts of Interest*, although this is not tobacco specific.
	4.2 Parties should formulate, adopt and implement a code of conduct for public officials, prescribing the standards with which they should comply in their dealings with the tobacco industry.	Met by general regulations and guidelines, including the *Staff Regulations* and the *Code of Conduct for Commissioners*, although these are not specific to tobacco.	Met in part by the *Code of Conduct for MEPs with Respect to Financial Interests and Conflicts of Interest*. However, this covers only financial COI and is not tobacco specific.
	4.3 Parties should not award contracts for carrying out any work related to setting and implementing public health policies with respect to tobacco control to candidates or tenderers who have conflicts of interest with established tobacco control policies.	No data.	No data.
	4.4 Parties should develop clear policies that require public office holders who have or have had a role in setting and implementing public health policies with respect to tobacco control to inform their institutions about any intention to engage in an occupational activity within the tobacco industry, whether gainful or not, within a specified period of time after leaving service.	Met by general regulations and guidelines, including the *Staff Regulations* and the *Code of Conduct for Commissioners*, although these are not specific to tobacco.	Met in part by the *Code of Conduct for MEPs with Respect to Financial Interests and Conflicts of Interest*, but there are no restrictions on subsequent employment during a “cooling off” period.
	4.5 Parties should develop clear policies that require applicants for public office positions which have a role in setting and implementing public health policies with respect to tobacco control to declare any current or previous occupational activity with any tobacco industry whether gainful or not.	Met by general regulations and guidelines, including the *Staff Regulations*, although these are not specific to tobacco.	Met in part by the *Code of Conduct for MEPs with Respect to Financial Interests and Conflicts of Interest*, although this is not tobacco specific
	4.6 Parties should require government officials to declare and divest themselves of direct interests in the tobacco industry.	Partially met by general regulations and guidelines, including the *Code of Conduct for Commissioners*, although these are not specific to tobacco.	Met in part by the *Code of Conduct for MEPs with Respect to Financial Interests and Conflicts of Interest* but this is not tobacco specific and no explicit reference is made to divestment.
	4.7 Government institutions and their bodies should not have any financial interest in the tobacco industry, unless they are responsible for managing a Party’s ownership interest in a State-owned tobacco industry.	No data.	No data.
	4.8 Parties should not allow any person employed by the tobacco industry or any entity working to further its interests to be a member of any government body, committee or advisory group that sets or implements tobacco control or public health policy.	Partially met by general measures in place to mitigate conflicts of interest in the appointment of external expert groups, but these are not tobacco specific. No data beyond this.	Partially met through general provisions in the *Code of Conduct for MEPs with Respect to Financial Interests and Conflicts of Interest*, but not tobacco specific. No data beyond this.
	4.9 Parties should not nominate any person employed by the tobacco industry or any entity working to further its interests to serve on delegations to meetings of the Conference of the Parties, its subsidiary bodies or any other bodies established pursuant to decisions of the Conference of the Parties.	No relevant data identified in relation to this. No formal policy on this identified.	N/A
	4.10 Parties should not allow any official or employee of government or of any semi/quasi-governmental body to accept payments, gifts or services, monetary or in-kind, from the tobacco industry.	Partially met by general regulations and guidelines, including the *Staff Regulations* and the *Code of Conduct for Commissioners*, although these are not specific to tobacco.	Met in part by the *Code of Conduct for MEPs with Respect to Financial Interest and Conflicts of Interest*, although this is not specific to tobacco.
	4.11 Taking into account national law and constitutional principles, Parties should have effective measures to prohibit contributions from the tobacco industry or any entity working to further its interests to political parties, candidates or campaigns, or to require full disclosure of such contributions	N/A	Met in part by the *Code of Conduct for MEPs with Respect to Financial Interest and Conflicts of Interest*. A full appraisal would require analysis of national political parties and campaigns, as well as at the EU level.
Require that information provided by the tobacco industry be transparent and accurate.	5.1 Parties should introduce and apply measures to ensure that all operations and activities of the tobacco industry are transparent.	Partially met through transparency initiatives but these are not comprehensive or tobacco specific.	Partially met through transparency initiatives but these are not comprehensive or tobacco specific.
	5.2 Parties should require the tobacco industry and those working to further its interests to periodically submit information on tobacco production, manufacture, market share, marketing expenditures, revenues and any other activity, including lobbying, philanthropy, political contributions and all other activities not prohibited or not yet prohibited under Article 13 of the Convention.	Partially met by the transparency register in relation to lobbying, but not comprehensive and not tobacco specific.	Partially met by the transparency register in relation to lobbying, but not comprehensive and not tobacco specific.
	5.3 Parties should require rules for the disclosure or registration of the tobacco industry entities, affiliated organizations and individuals acting on their behalf, including lobbyists.	Partially met by the transparency register in relation to lobbying, but not comprehensive and not tobacco specific.	Partially met by the transparency register in relation to lobbying, but not comprehensive and not tobacco specific.
	5.4 Parties should impose mandatory penalties on the tobacco industry in case of the provision of false or misleading information in accordance with national law.	No relevant data identified.	No relevant data identified.
	5.5 Parties should adopt and implement effective legislative, executive, administrative and other measures to ensure public access to a wide range of information on tobacco industry activities as relevant to the objectives of the convention, such as in a public repository.	Partially met by Regulation 1049/2001 on access to information by the public, including meetings with officials, but places the burden on the public rather than the Commission and may provide partial (redacted) information.	Partially met by Regulation 1049/2001 on access to information by the public, but places the burden on the public and may provide partial (redacted) information.

^a^As with the article more generally, this table focusses on certain guidelines which are most relevant to the focus of our analysis. Guidelines 3.1–3.4, 6.1–6.4, 7.1–7.3 and 8.1–8.3 are thus excluded from the table. Where there is an absence of relevant data to evaluate compliance with a guideline we state this. Similarly, where relevant guidelines cannot be applied to a supranational entity such as the EU (versus a state actor), or cannot be applied for any other reason, we mark these ‘not applicable’ (N/A)

^b^ Analysis of the European Parliament here is limited to MEPs, as opposed to permanent officials in the Parliament Secretariat

The apparent reluctance to implement Article 5.3 guidelines fully suggests a prioritisation of business interests over public health within the EU institutions, a finding that is consistent with previous research [8, 50]. For some Commission officials, in particular, tobacco control was not seen as a political priority which warranted the measures to curtail tobacco industry influence contained within Article 5.3. Instead, it was feared that restrictions on engagement with TTCs may be the “thin end of the wedge” which would lead to similar measures being introduced to curtail access to officials by commercial, and potentially other, civil society actors. This desire to balance health imperatives against the interests of businesses reflects that seen in other contexts, where non-health ministries often see government-industry interaction as normal and are unwilling to alter their practices in relation to the tobacco industry [5, 6, 51]. This underlines both the importance of international conventions like the FCTC in creating a rationale for excluding TTCs from policy making and the importance of signatories implementing explicit policies and procedures to give effect to them. Experience elsewhere suggests that establishing a core group or committee under the auspices of the Ministry of Health, with representation from relevant NGOs, to oversee the implementation of Article 5.3 can be an effective means of overcoming industry obstructionism and the institutional inertia of non-health ministries [6, 52].

Finally, there appears to be a clear hierarchy of legal norms within EU institutions, which prioritises EU law over the EU’s obligations as a Party to international treaties such as the FCTC. The issue of Article 5.3 compliance presents an example of where the EU’s foundational treaties are perceived by some actors to come into conflict with its international treaty obligations under the FCTC, e.g. the apparent lack of an EU treaty basis for Article 5.3 measures relevant to MEPs. While the independence of elected representatives is an important issue that raises difficult questions in the context of Article 5.3 implementation, this hierarchy of legal norms stands in contrast to the primacy of EU law over national laws and the requirement of member states to bring domestic regulations into line with EU law. It also contrasts with the generally accepted binding nature of international trade and investment law and the requirement of states and Parties such as the EU to implement the commitments this places on them, even where this curtails the Party’s policy space [17]. It is not clear why an international agreement to protect public health should be treated differently. In its response to the Ombudsman, the Commission asserted that, while Article 5.3 (and the FCTC more broadly) was legally binding, the WHO’s Article 5.3 guidelines were not [43]. However, it has been argued that, as a document adopted by the COP, the guidelines constitute a subsequent agreement under Article 31 of the Vienna Convention on the Law of Treaties 1969, and should thus be taken into account when interpreting FCTC Parties’ treaty obligations [3, 53]. While the wording of the preamble to the guidelines suggests that they are non-binding, they also “strongly urge” Parties to “implement measures beyond those recommended in these guidelines when adapting them to their specific circumstances” [2]. Finally, we note that, following the entry into force of the Treaty of Amsterdam in 1999, the EU is required to “ensure a high level of human health protection” in all its policies and activities [54].

## Conclusion

It is clear that EU institutions have some way to go in order to properly implement the requirements of Article 5.3. While there is growing awareness of the issue in both the Commission and the EP, in large part as a result of the experience of lobbying over the TPD, there is also considerable resistance to further substantive action to implement it in both institutions, including at the highest levels of the institutions. Article 5.3 implementation requires much more to be done to institutionalise rules and practices guarding against the influence of the tobacco industry. As the EU Ombudsman noted in her decision on the inquiry into the Commission’s implementation of Article 5.3, the inquiry provided the Commission with an opportunity to build on its experience of industry lobbying over the TPD to set “a global benchmark for compliance with Article 5.3” [42]. It has so far failed to set such an example. While EU institutions have pre-existing guidelines and practices that go some way to meeting the requirements of Article 5.3 – such as rules and guidelines on transparency and the avoidance of conflicts of interest – these are not specifically designed to meet the requirements of Article 5.3 and leave substantial gaps, which can be exploited by tobacco companies and their proxies. More research is needed on Article 5.3 compliance in other EU institutions and agencies not explicitly examined here.

On the basis of the results discussed in this article, we recommend that a binding and comprehensive policy and code of conduct specifically designed for the implementation of Article 5.3, and based on the WHO’s guidelines, be created to cover the activities of all employees of the EU institutions. Crucially, such a code would need to deal explicitly with third parties acting for the tobacco industry. Article 5.3 compliance measures and practices currently in place within DG Santé (formerly Sanco) are the most advanced of any EU body, despite their limitations, and may provide a model for other bodies to follow in the first instance. DG Santé, or other relevant entities within the Commission, should also consider the convening of a core group or committee with a specific remit to examine the implementation of Article 5.3 across the EU institutions, in consultation with relevant NGOs, health experts and other EU bodies. The need for proper awareness raising, among MEPs and officials, is abundantly clear. While the distinction between elected representatives and appointed functionaries is an important one, the EP should discuss, as a matter of urgency, how Article 5.3 can be implemented in a manner consistent with MEPs’ mandate.

## Abbreviations

AMCHAM: The American Chamber of Commerce
BDI: Bundesverband der Deutschen Industrie (The Federation of German Industry)
CCC: Code of Conduct for Commissioners
CEO: Corporate Europe Observatory
CETA: Comprehensive Economic and Trade Agreement
CJEU: Court of Justice of the European Union
COI: Conflict of Interest
COP: Conference of the Parties
DG: Directorate General (of the European Commission)
EAEC: European Atomic Energy Community
EEC: European Economic Community
ENVI: Environment Public Health and Food Safety Committee of the European Parliament
EP: European Parliament
EPP: European People’s Party
EU: European Union
FCTC: Framework Convention on Tobacco Control
FOI: Freedom of Information
MEP: Members of the European Parliament
S&D: Progressive Alliance of Socialists and Democrats
TPD: Tobacco Products Directive
TTC: Transnational Tobacco Company
TTIP: Trans-Atlantic Trade and Investment Partnership
WHO: World Health Organization
